# Usefulness of the Adipokines as Biomarkers of Ischemic Cardiac Dysfunction

**DOI:** 10.1155/2018/3406028

**Published:** 2018-10-10

**Authors:** Larisa-Diana Mocan Hognogi, Cerasela-Mihaela Goidescu, Anca-Daniela Farcaş

**Affiliations:** ^1^Internal Medicine Department, “Iuliu Hațieganu” University of Medicine and Pharmacy, Cluj-Napoca, Romania; ^2^Emergency Clinical County Hospital, Cluj-Napoca, Romania

## Abstract

Cardiovascular disease is the leading cause of death among both women and men, but there is still a great percentage of misdiagnosis and lack of clearly defined criteria. Advances in biomolecular science have proven the crucial role of inflammation and, more importantly, the role of adipokines in mediating all stages of coronary artery disease. It has also been suggested that regional fat deposits, more precisely from thoracic region, have a major influence on the development of coronary artery disease by creating a local proatherogenic environment. The immune system closely interacts with metabolic risk factors to initiate, promote, and further aggravate the atherosclerotic lesions on the arterial wall all with the “help” of adipokines. So nowadays, research extensively focuses on uncovering biomarkers that would provide an increased chance of detecting subclinical cardiac distress and also add a consistent value to current guideline-imposed risk criteria.

## 1. Introduction

**Figure 1:**
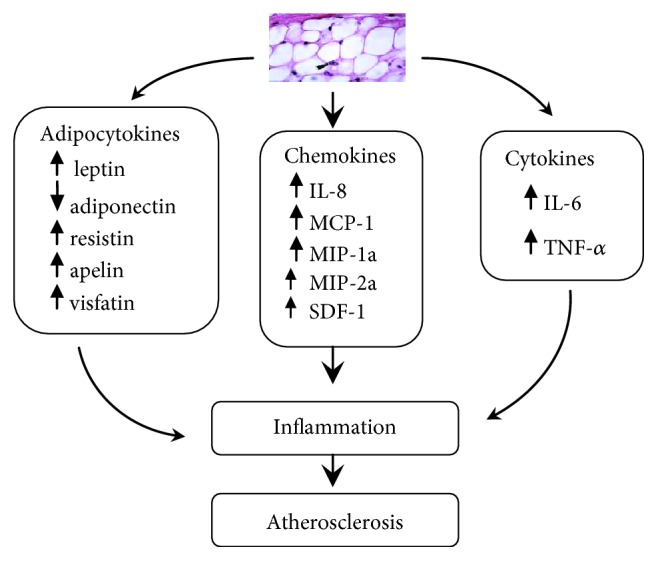
The role of obese adipocytes in the inflammation and pathogenesis of atherosclerosis (adapted from Opatrilova et al. [4]).

The adipose tissue secretes several hormone-like molecules called adipokines, which participate in body physiology regulation. Adipokines, pleiotropic molecules, also play a role in diseases such as diabetes, atherosclerosis, and autoimmune diseases, among others [1]. The adipokine group includes classical cytokines (e.g., tumor necrosis factor-*α* (TNF*α*), interleukin-6 (IL-6)), specific chemokines (interleukin-8 (IL-8), monocyte chemoattractant protein-1 (MCP-1), macrophage inflammatory protein-1*α* (MIP-1*α*), macrophage inflammatory protein-2*α* (MIP-2*α*), stromal cell-derived factor-1 (SDF-1)), growth factors (e.g., transforming growth factor-*β* (TGF-*β*)), and proteins of the alternative complement system (e.g., adipsin, acylation-stimulating protein). The group also includes proteins involved in vascular hemostasis (e.g., plasminogen inhibitor activator-1 (PAI-1), tissue factor), lipid metabolism (leptin, retinol-binding protein, and cholesteryl ester transfer protein), glucose homeostasis (e.g., adiponectin, resistin), and angiogenesis (e.g., vascular endothelial growth factor (VEGF)), as well as acute phase and stress responses (e.g., haptoglobin, metallothionein) [2, 3]. Production of these proteins by adipose tissue is increased in obesity and has led to the idea that obese patients are characterised by a state of chronic low-grade inflammation, that links causally to insulin resistance and the metabolic syndrome.  Figure 1 describes the role of obese adipocytes in the inflammation and pathogenesis of atherosclerosis [4].

There is no doubt that inflammation is viewed as an important pathophysiological step in the development of atherosclerosis. There are a multitude of studies that address this issue and also the issue of the interaction between the immune mechanisms and metabolic risk factors. This interaction initiates, promotes, and activates the lesions in the coronary arteries, and currently, efforts are made to find the key biomarkers that could unveil the risk of the progression of atherosclerosis in patients with metabolic disorders.

## 2. Adipokines as Biomarkers in Atherosclerotic Heart Disease

### 2.1. Biomarker Definition

**Figure 2:**
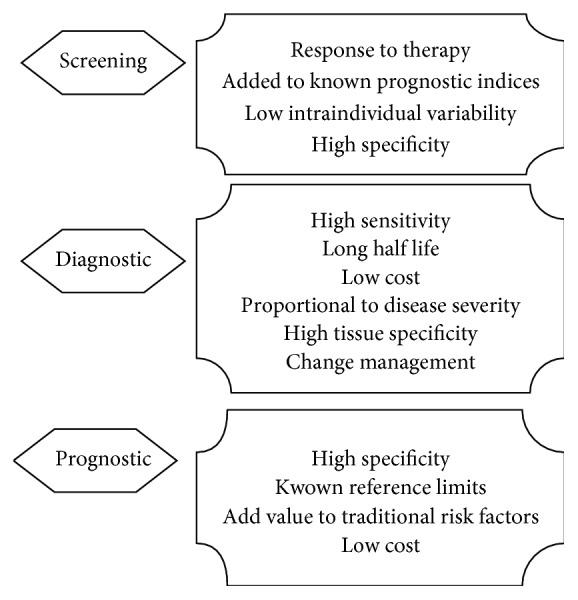
Ideal biomarker characteristics (adapted from Zhao et al. [9]).

A biomarker is a “characteristic that is objectively measured and evaluated as an indicator of the normal biological process, pathogenic processes, or pharmacologic responses to a therapeutic intervention” [5]. An ideal biomarker should be used for screening, prognostic, and diagnostic purposes (Figure 2).

Recent research has shown that medicine needs precision, and it is important to have in mind that people are different with respect to genes, environment, and lifestyle factors and that treatments need to be more effective and targeted. This is one of the reasons that we need prognostic, pharmacodynamic, or predictive biomarkers [6, 7].

When introducing a new biomarker, one has to keep in mind that this novel molecule needs to be as close as possible to an “ideal biomarker” regarding accuracy and standardization of determination, reproducibility, accessibility, high sensibility, and specificity and it should also make an impact in the clinical care of a patient [8].

When interpreting the serum levels of a biomarker, one should refer to the following:

- Reference limits: these are cut-off values statistically established taking into consideration disease-free individuals
- Discriminative limits: these are the limits that impose a decision. For example, if we take into account troponin, the 99 percentile is discriminative for acute myocardial infarction so that we can correctly assess patients with acute myocardial infarction apart from healthy individuals
- Risk limit: this is the limit from where we consider a greater risk

At the present time, there are only a few number of established biomarkers (e.g., NT-proBNP, BNP, cTnI, cTnT, CRP, and D-dimers) but important research is being conducted in developing new ones as the diagnostic, prognostic, and therapeutic impact is remarkable.

### 2.2. Classification of Biomarkers in Cardiovascular Disease

There is a large number of biomarkers evaluated or under evaluation in relation with cardiovascular disease, and detailing all of them exceeds the purpose of this paper. But, if we strictly refer to atherosclerotic disease, they can be grouped as follows [6, 10]:

- Biomarkers for acute changes: copeptin, high sensitivity troponin, galectin-3, ST-2, heart fatty acid-binding protein (H-FABP), pregnancy-associated plasma protein-A (PAPP-A), ischemia-modified albumin (IMA)
- Biomarkers for chronic changes: coronary calcium
- Biomarkers of inflammation: C-reactive protein, interleukin-6, fibrinogen, monocyte chemotactic protein-1, TNF-alpha, myeloperoxidase, soluble fragment CD40 ligand (sCD40L), angiotensin II, E-selectin, heat shock proteins, matrix metalloproteinases (MMP), myeloperoxidase (MPO), platelet endothelial cell adhesion molecule-1 (PECAM-1), intracellular cell adhesion molecule 1 (ICAM-1), vascular cell adhesion molecule 1 (VCAM-1)
- Biomarkers of metabolic disorders: lipoprotein (a), low-density lipoproteins, high-density lipoproteins, apoB100, lipoprotein-associated phospholipase A2, homocysteine, adiponectin, haptoglobin, visfatin, leptin, resistin, etc.

Some of the above listed are already documented as biomarkers, so the present paper refers to those that derive from the adipose tissue and that are still under research but with promising results, which we have also confirmed in our studies.

#### 2.2.1. Visfatin in Atherosclerosis

Visfatin is a new identified adipokine that plays a role as a proinflammatory mediator in the process of atherosclerosis and also in plaque destabilization, also known as NAMPT (nicotinamide phosphoribosyltransferase) and PBEF (pre-beta cell-enhancing factor). Research has shown that this adipokine has insulin mimetic features and is involved in the process of pancreatic pre-beta cell maturation. It was first described in 2005 by Fukuhara and his collaborators [11]. These authors showed that visfatin correlates with visceral fat. Even though their research paper was partially retracted due to technical issues, mainly, the multiple functions of this cytokine were confirmed in an increasing number of studies. Also, it was demonstrated that bariatric surgery in morbidly obese patients helped lower the plasma level of visfatin 6 months afterwards [12]. Obesity and T2DM are independent risk factors for atherosclerotic disease, and it seems that plasma levels of visfatin are increased in this set of subjects. Kadoglou et al. demonstrated this in 2010, in a study conducted on 120 diabetic patients without clinical vascular complications, compared with age- and sex-matched healthy individuals [13].

Visfatin acts on a large number of sites as follows [14]:

- Endothelial wall: angiogenesis, tumor growth, cardiac fibrosis
- Cardiomyocytes
- Vascular tone: in healthy subjects, it promotes vasodilation, but in subjects with T2DM, it promotes vasoconstriction
- Extracellular matrix: it determines augmentation in MMP2/9 release which promotes plaque weakening

As it is well known by now, arterial hypertension, dyslipidemia, smoking, and T2DM determine repetitive lesions on the endothelium of the arterial vessels. This leads to a growing inflammatory response. There are a number of authors that have demonstrated that in the presence of inflammation, visfatin levels are increased. Visfatin may contribute to cardiac fibrosis indirectly via the vascular endothelium and acts at this level (of the endothelium) by superactivating ERK-NT-kB-iNOS axis, thus activating among others, matrix metalloproteinases. Visfatin also enhances the production of myocardial repair tissue (when cardiomyocytes were acutely exposed to it) and remodeling (if cardiomyocytes were chronically exposed to it) via upstreaming matrix metalloproteinase-9 and vascular endothelial growth factor [15]. This is confirmed by demonstrating the superexpression of mRNA. Also, eNAMPT enhances cardiac hypertrophy via calcineurin/NFAT signaling pathway—a genetic mechanism involved in cardiac hypertrophy. Visfatin is also known as NAMPT (nicotinamide phosphoribosyltransferase), and NAMPT has the role as an NAD+-synthesizing enzyme within the cell (iNAMPT). This enzyme protects against cardiac hypertrophy by maintaining optimal high levels of NAD+. But when released into the circulation, eNAMPT acts as a proinflammatory adipokine, cytokine, and growth factor and induces endothelial dysfunction and destabilization of atherosclerotic plaque [16].

Therefore, in concern to the involvement of visfatin in the atherosclerotic plaque, this is due to the fact that it acts like an intracellular regulator of NAD+-dependent reactions in the muscle cell. It also promotes vascular inflammation and proliferation in association with atherosclerotic plaque [17].

Until now, the majority of research showed that visfatin might be in relation with obesity but mostly in the presence of T2DM. Hypertension does not seem to alter visfatin plasma levels. Our study, conducted on two different types of hypertensive patients, also confirms these findings [18].

If we were to analyze visfatin, from a biomarker view, one notices that, at this moment, it seems to meet some of the criteria mentioned before:

- it has high specificity: values to be found elevated in T2DM with obesity
- it adds value on traditional risk: patients with obesity and T2DM who also had high visfatin plasma levels seem to have more advanced atherosclerosis

With respect to the response to therapy, it was found that lipid-lowering therapy significantly lowers visfatin plasma levels. Also, there are a number of studies that show that inhibiting visfatin might represent a novel therapeutic approach in cardiovascular or cerebrovascular complications of atherosclerosis [19].

#### 2.2.2. Apelin in Atherosclerosis

Apelin is a relatively newly discovered peptide, with various and significant activities within the human body. Initially, it was considered to be an adipocytokine, but it was concluded that its most important actions are directed to the cardiovascular system particularly in the endothelium and myocardium. Both apelin and its receptor, APJ, are highly expressed in the vessels and heart. It is synthetized as preprohormone and then cleaved in fragments of different lengths [20–23]. The majority of its effects are chain links in a complicated and intricate protection mechanism which is designed to regulate basic functions of the organism, aid the regenerative processes, and slow the diseases. It is subject for research in various fields and pathologies, the researchers' attention being focused on apelin-13, the most important apelin isoform for the cardiovascular system [24]. Its protective activity is mainly accomplished through antagonism of the renin-angiotensin-aldosterone system, but other mechanisms are revealed in the latest research [25–28].

This protective effect of apelin is expressed also on the atherosclerotic process and its development [29, 30]. Apelin-13 inhibits the formation of the macrophage foam cells; the main mechanism for that is the activation of the protein kinase C and initiation of some molecular pathways that in the end will significantly reduce the cholesterol level in the macrophage foam cells and the production of foam cells themselves [31]. In this process, the stimulation of autophagy is the key effect [32]. In favor of this result came the observations of Cui et al. about microRNAs; their increased expression has an interesting effect of reducing the expression of apelin in macrophages concomitant with increasing the lipid accumulation and lowering the efflux of cholesterol from macrophages. At the same time, the apelin molecules that were resistant to microRNA effects continued to inhibit the accumulation of lipids in the macrophages. According to Cui et al. observations, miRNA-497 is involved in modulating the efflux of oxidized LDL cholesterol from the THP-1 macrophages and apelin is one of the molecule from the downstream molecular chain reactions. Overexpression of miRNA-497 was observed to significantly reduce the expression of apelin in THP-1 macrophages [33]. Enforced expression of miR-497 promoted lipid accumulation and decreased cholesterol efflux in oxLDL-exposed THP-1 macrophages. In contrast, downregulation of miR-497 suppressed oxLDL-induced lipid accumulation in THP-1 macrophages. Overexpression of miR-497 significantly reduced the expression of apelin in THP-1 macrophages. The levels of microRNA studied in this research were increased and negatively correlated with the level of apelin in the atherosclerotic lesions [34].

The experimental studies showed that the administration of apelin-13 results in lower levels of proinflammatory cytokine secretion (interleukin-6, interleukin-1*β*, and tumor necrosis factor-*α*) and lower expression of lipoprotein lipase, and so, it favors the reduction of lipid accumulation in the vascular wall [35]. In experimental models of aortic abdominal aneurysm induced by elastase, the exogen apelin administration reduces the content of macrophages within the aortic wall and the chemokine production and in the end, reduces the formation of aortic aneurysm [36].

The initial studies showed that in patients with acute coronary syndromes, the level of apelin lowered as the severity of coronary stenosis increased. Highly unstable atherosclerotic plaques were associated with lower levels of apelin [37, 38]. Furthermore, this peptide appears to be involved in the development of collateral vessels in patients with stable angina; the study of Akboga et al. showed that apelin can be a predictor of collateral vessel development, besides other factors such as the severity of stenosis or the presence of right coronary occlusion or severe multivessel disease. In their research, apelin was an independent predictor of good coronary collateral network.

Although the data is still incomplete, apelin appears to be an atheroprotective factor, and most important, it shows potential for therapeutic manipulation but also for risk stratification. But further studies are needed to clarify both the predictive value of this marker and its therapeutic value.

Atherosclerosis is a major pathology which affects all vital organs and is a cause for severe morbidity and mortality, with increased costs. Any therapeutic modulation of the pathophysiologic process in order to limit the clinical consequences is welcomed, and apelin appears to be an important potential target for treatment. Another potential target could be the adipose tissue macrophage activation as suggested by the paper of Boutens et al. [39].

#### 2.2.3. Leptin in Atherosclerosis

Through the adipocytokines released by the fatty tissue, after a unique metabolic rewiring, the fat tissue influences the inflammation in the whole body, particularly the atherosclerotic lesion development [40]. Leptin is an adipocytokine produced in the adipose tissue with a primary role of regulating the food intake and energetic metabolism, in order to keep the fat tissue at a constant level. It is an anorectic agent through actions on the hypothalamus; because obese patients have a high level of serum leptin, it was thought that the main mechanism in obesity is a resistance to leptin [41].

This adipocytokine has also an important activity on the cardiovascular system. It was shown that it is both atherogenic and antiatherogenic factors, and it appeared to be a predictive marker for cardiovascular events. Its importance as a protective factor is not yet clarified, although it was suggested that hyperleptinemia might not be directly linked to atherogenesis, but it might reflect and be a consequence of a state of leptin resistance. The obesity paradox was described, and it represents only a small part of the complex molecular system that controls and promotes the inflammation and its effect on blood vessels [42–44].

It appears that high levels of leptin promote the production of other inflammatory mediators such as tumor necrosis factor, interleukin-2, and interleukin-6 and increase the production of the reactive oxygen radicals. Leptin stimulates the proliferation and hypertrophy of smooth muscle cells within the vessel wall and the accumulation of cholesterol esters in foam cells, particularly if associated with hyperglycemia [42, 45]. It is well known that all these effects contribute to endothelial dysfunction and promote the development and progression of atherosclerotic lesions, but it is not yet clear if leptin itself can induce the atherosclerotic process or it is only a marker of the biological context in which the vascular lesions are produced.

Leptin was intensely studied in cardiovascular patients, and it was observed that it is predictive for metabolic syndrome, myocardial infarction and coronary events in men and hypertensive women, and ischemic stroke [42, 46]. In combination with adiponectin, another adipocytokine with protective effects for the vessels, leptin is a valuable marker for the prediction of the atherosclerotic process, both their serum values being useful, but more importantly their ratio (leptin/adiponectin) [47].

Leptin, together with adiponectin, is a link in the adipovascular axis, connecting the excessive fat tissue with inflammation and atherosclerosis, and all the pathologies that derive from their imbalance. The leptin/adiponectin ratio is a new and better marker for atherosclerosis (and for monitoring the atherosclerotic index), insulin resistance, and endothelial dysfunction, but gender differences must be kept in mind because of the hormonal influences on the adipose metabolism and adipokine production [47].

#### 2.2.4. Resistin in Atherosclerosis

Human resistin, a 12.5 kDa cysteine-rich polypeptide [48] circulating in different molecular isoforms [49], is expressed at lower levels in adipocytes but at higher levels in circulating monocytes and macrophages [50] and vascular endothelium [51]. It has proinflammatory effects by nuclear factor-kappa B (NF-*κ*B) activation and production of cytokines (IL-6, IL-1, TNF-alpha, and monocyte chemoattractant proteins) [52, 53]. At the same time, it exerts vascular remodeling effects by the stimulation of angiogenesis [54], proliferation capacity [55], and expression of endothelin-1, VCAM-1, and MCP-1 [56] by vascular smooth muscle cells. Resistin also decreases the production of nitric oxide in coronary endothelial cells and increases the production of reactive oxygen species (ROS) [57].

As a result, resistin stimulates monocytes, endothelial cells, and vascular smooth muscle cells, thereby inducing atherosclerosis in experimental animals [58]. These experimental evidences, together with clinical studies demonstrating the association of resistin plasma levels with obesity [59, 60], metabolic syndrome (MetS) [61], and ischemic heart disease [62, 63], suggest that resistin might play a role in the interaction between insulin resistance, inflammation, and atherosclerosis [59, 60]. Several studies have shown that variants of the resistin gene and their imbalance have an impact on metabolic parameters and cardiovascular risk [64, 65]. Subjects with 420C/G allele showed increases in resistin plasma concentrations [66, 67], elevated glucose levels at birth [68], high HbA1c levels [69], and increased risk of T2DM [65]. At the same time, they also had increased levels of triglycerides and higher prevalence of MetS and obesity [61]. In both females and males, the G allele of the 420C/G of the resistin gene polymorphism appears to be associated [70] with higher risk for cardiovascular events [61], cerebrovascular disease, and stroke [69, 71]: more severe stroke and higher in-hospital mortality in patients with acute ischemic stroke [72]. Similarly, subjects with allele 299A (allele +299 (G>A) alleles) exhibited high glucose levels at birth, and in diabetic patients, the resistin levels correlated with cerebrovascular disease especially in males [72].

Although these characteristics make resistin a good biomarker of atherosclerosis, its relationship with IMT—a marker of initial asymptomatic atherosclerosis—has been reported only in obese children [73] but there is no evidence of this association in obese adults [74]. In contrast, in obese patients, resistin correlated with key components that correlate vascular risk parameters to the development of atherosclerosis: platelet number and volumes (MPV), serum and platelet P-selectin, fibrinogen, PAI-1 ag (plasminogen activator inhibitor-1 antigen), and PMPs (platelet-derived microparticles [16, 46, 74]). At the same time, increased resistin levels found in smokers correlate with insulin resistance, while both in smokers and nonsmokers, they are not related to C-reactive protein, homocysteine, and uric acid levels [12, 46, 70].

In addition, patients with premature coronary atheromatosis (diagnosed on coronary angiogram or with acute infarction before 45 years) showed elevated serum levels of resistin compared to those without, in the presence of the same risk factors (even in the absence of significant differences between risk factors) [75]. Moreover, in a cohort of patients, resistin has been shown to be an independent predictor of acute coronary syndromes (ACS) (in patients without a history of myocardial infarction or stroke) and for cardiac or cerebrovascular events (in patients with coronary artery disease [63]). Patients with unstable angina and acute myocardial infarction (STEMI or NSTEMI) have elevated serum levels of resistin, suggesting a possible role as a diagnostic biomarker for acute coronary events [76, 77]. Resistin levels increase starting at 3–6 hours after the onset of chest pain and peaked at 12 hours. In an average follow-up of 83.4 months, resistin levels were an independent predictor of a new acute ACS [78, 79] in patients with coronary artery disease [80]. In patients with stable angina treated by PCI, resistin levels increased at 12 hours after the procedure but did not correlate with the increases in troponins levels [81].

The relationship of resistin with the risk of stroke requires a further study. One study on postmenopausal women found an association between increased resistin levels and the risk of stroke, regardless of the presence of obesity and other risk factors for cardiovascular disease [82], a conclusion not confirmed by another large study [83]. Serum resistin levels were able to predict mortality at 5 years and functional outcomes in patients with ischemic stroke [84, 85]. Studies have also identified resistin levels in patients with overt peripheral atherosclerosis. In one study, these were elevated in patients with peripheral arterial disease (PAD) compared to those with ischemic heart disease [86] and plasma levels of resistin and diastolic BP were predictors of new ischemic events and readmission for nonfatal myocardial infarction, heart failure, or critical limb ischemia in these patients. Moreover, in patients with PAD who underwent bypass surgery, increased levels of resistin were able to predict the reduced free survival range without amputation in patients with critical limb ischemia, regardless of the presence of diabetes [87].

## 3. Conclusion

Nowadays, we are facing a tremendous amount of information that puts us, as practitioners, in front of many decisions that have to be as accurate as possible. Research in the last decades proved that obesity is extremely bound to systemic inflammation and that visceral adiposity increases cardiovascular risk, namely, the risk of cerebrovascular disease and myocardial infarction. Chronic inflammation is linked to endothelial dysfunction and elevated prothrombin activity, and therefore, there is still a need to evaluate the impact of the adipokines in relation to various clinical outcomes of atherosclerosis so that these molecules could become great tools to predict the dynamics and therapeutic response of the disease.
